# Peripheral precocious puberty in girls with McCune-Albright syndrome: a case series

**DOI:** 10.20945/2359-4292-2024-0459

**Published:** 2025-04-27

**Authors:** Aline Guimarães Faria, Luciana R. Montenegro, Alexander Augusto Lima Jorge, Regina Matsunaga Martin, Maria Candida Barisson Villares Fragoso, Flavia R. Tinano, Carlos E. Seraphim, Ana Pinheiro Machado Canton, Larissa G. Gomes, Gabriel A. Martos-Moreno, Irene Tarjuelo García, Atilano Carcavilla, Mireia Tirado-Capistros, Nadja Cristhina Souza-Pinto, Jesús Argente, Ana Claudia Latronico, Berenice Bilharinho Mendonca, Vinicius Nahime Brito

**Affiliations:** 1 Unidade de Endocrinologia do Desenvolvimento, Laboratório de Hormônios e Genética Molecular LIM/42, Hospital das Clínicas, Faculdade de Medicina, Universidade de São Paulo, São Paulo, SP, Brasil; 2 Unidade de Endocrinologia Genética, Laboratório de Endocrinologia Celular Molecular LIM/25, Disciplina de Endocrinologia, Faculdade de Medicina, Universidade de São Paulo, São Paulo, SP, Brasil; 3 Unidade de Adrenal e Desenvolvimento, Laboratório de Hormônios e Genética Molecular LIM/42, Divisão de Endocrinologia e Metabologia, Hospital das Clínicas, Faculdade de Medicina, Universidade de São Paulo, São Paulo, SP, Brasil; 4 Departamento de Pediatría y Endocrinología Pediátrica, Hospital Infantil Universitario Niño Jesús, Instituto de Investigación La Princesa; CIBER Fisiopatología de la Obesidad y Nutrición (CIBEROBN), Instituto de Salud Carlos III, Madrid, Spain; 5 Servicio de Endocrinología Pediátrica, Hospital Universitario Sanitas La Moraleja, Madrid, Spain; 6 Departamento de Endocrinología Pediátrica, Skeletal Dysplasia Multidisciplinary Unit (UMDE-ERN BOND), European Research Network on Rare BONe Disorders (ERN-BOND), Hospital Universitario La Paz, Madrid, Spain; 7 Servicio de Pediatría, Hospital de la Santa Creu i Sant Pau; Departamento de Pediatría, Obstetricia y Ginecología y Medicina Preventiva y Salud Pública; Universitat Autònoma de Barcelona, Institut de Recerca Biomèdica Sant Pau (IIB-Sant Pau), Barcelona, Spain; 8 Laboratório de Genética Mitocondrial, Departamento de Bioquímica, Instituto de Química, Universidade de São Paulo, São Paulo, SP, Brasil; 9 IMDEA, Food Institute, CEIUAM+CSI, Cantoblanco, Madrid, Spain; 10 Laboratório de Sequenciamento em Larga Escala (SELA), Faculdade de Medicina, Universidade de São Paulo, São Paulo, SP, Brasil

**Keywords:** McCune-Albright syndrome, precocious puberty, aromatase inhibitors, tamoxifen, digital droplet PCR

## Abstract

**Objective:** To describe the follow-up of girls with peripheral precocious puberty (PPP) due to McCune-Albright syndrome (MAS). **Subjects and methods:** Data from 18 females, including anthropometric and reproductive outcomes, were evaluated. Genetic analysis was performed on DNA from peripheral leukocytes via digital PCR. **Results:** Clinical manifestations of PPP were isolated thelarche, thelarche plus vaginal bleeding, or isolated vaginal bleeding in 44.5%, 33.3%, and 22.2%, respectively, at an early age (3.3 ± 1.6 years). At diagnosis, basal LH and FSH levels were suppressed in 100% and 72.2% of cases, respectively, and estradiol ranged from prepubertal to high levels. The mean bone age advancement was 2.3 ± 1.9 years. Treatment included medroxyprogesterone acetate, tamoxifen, aromatase inhibitors, and ketoconazole, individually or in combination for 5 ± 2.14 years, with partial or complete control of puberty. Secondary central precocious puberty was diagnosed in 57.1% (8/14) of the patients. Fibrous dysplasia was diagnosed in 11 patients and managed with bisphosphonates for those with bone pain. The mean adult height was 155.1 ± 8.7 cm (-1.17 SDS) in 10 patients. Menarche occurred at a mean age of 12.2 ± 1.04 years, 70% reported regular menstrual cycles and only one female desired pregnancy. Genetic diagnosis was established in 52.9% (9/17) of the patients. **Conclusion:** Medical treatment of PPP was effective in girls with MAS and led to preservation of adult height potential, and reproductive function was normal when patients reached adulthood

## INTRODUCTION

First described in 1937 (^1^), McCune-Albright syndrome (MAS) is now known to be caused by postzygotic mutations at codon 201 of the *GNAS* gene on chromosome 20q13.3, which leads to somatic activation of the stimulatory α-subunit of G protein (Gsα) (^2^,^3^). Due to its mosaic pattern, MAS manifests as a heterogeneous condition with a broad spectrum of clinical findings resulting from the variable tissue distribution of the *GNAS* mutation (^4^).

Peripheral precocious puberty (PPP) of ovarian origin represents one of the milestones of the classical triad of McCune-Albright syndrome (MAS) (^5^), which also includes fibrous dysplasia (FD) and *café-au-lait* skin pigmentation (^6^).

The diagnosis of MAS relies primarily on clinical assessment and is further confirmed by laboratory tests and imaging procedures (^6^). The genetic diagnosis of MAS is limited, mainly due to the mosaic distribution of *GNAS*-activating mutations that are frequently not detectable in peripheral blood (^7^). Very sensitive techniques, such as droplet digital PCR (ddPCR), have been proposed as promising methods to improve sensitivity in the genetic diagnosis of MAS using peripheral blood (^8^).

In MAS, PPP results from autonomous ovarian activation, which triggers early vaginal bleeding and breast development, and accelerates growth usually before age 4 (^4^). Estrogen levels vary widely, whereas gonadotropin levels remain at prepubertal levels or are suppressed. Secondary CPP may occur after prolonged estrogen exposure, particularly in patients with advanced bone age (^9^,^10^). Pelvic ultrasound aids in the detection of ovarian cysts (^6^). Clinical diagnosis relies on skin examination for hyperpigmentation and bone scanning for fibrous dysplasia lesions, while laboratory tests are crucial for the identification of associated endocrine disorders (^4^).

Treatment of progressive PPP in McCune-Albright syndrome (MAS) has historically been challenging (^11^). Previous options, such as progestin agents, antiandrogens (*e.g.*, cyproterone acetate), and steroidogenesis inhibitors (*e.g.*, ketoconazole), had limited efficacy and potential side effects (^11^). More recently, options such as tamoxifen, aromatase inhibitors, and estrogen receptor antagonists (e.g., fulvestrant) have emerged (^11^,^12^). Long-acting GnRH analogs are added to the treatment regimen if secondary CPP occurs (^4^,^13^). Combinations of these agents, which have complementary mechanisms, are common in clinical practice (^3^,^4^). However, their efficacy, alone or combined, is variable and debated, with concerns about long-term safety in pediatric patients (^6^,^14^,^15^). Aromatase inhibitors (*e.g.*, letrozole) have been increasingly recommended as first-line treatments, as they have demonstrated efficacy and safety in a large patient cohort with long-term follow-up (^15^).

Given the rarity of MAS, the ability to acquire personal experience is difficult, and since genetic diagnosis is rare, treatment approaches are highly significantly varied. Therefore, our objective was to comprehensively delineate a cohort of 18 female patients with MAS and PPP and to outline their clinical, laboratory, and imaging features at the time of diagnosis. Furthermore, we evaluated the therapeutic aspects of PPP resulting from MAS and examined long-term anthropometric and reproductive outcomes.

## SUBJECTS AND METHODS

The Ethics Committee of the University of Sao Paulo, Brazil, approved the study protocol. The parents/legal guardians of all the participants aged under 18 years provided written informed consent, and the children provided consent when applicable.

This was a retrospective, observational and longitudinal study that included 18 patients with PPP and MAS: fourteen consecutive patients (patients 1 to 14) were followed in the Endocrinology Outpatient Unit of the *Hospital das Clínicas da Faculdade de Medicina da Universidade de São Paulo*, Sao Paulo, Brazil, between 1980 and 2021, and four selected Spanish patients (patients 15 to 18) were followed in Spanish tertiary pediatric centers. The inclusion criteria were PPP of ovarian origin before 8 years of age and at least one other clinical feature of MAS.

The medical records of all patients were systematically reviewed. The following data were documented at diagnosis: chronological age (CA) at the first pubertal sign (thelarche or vaginal bleeding), CA at the first medical evaluation, height (cm/SDS), pubertal stage (Tanner’s criteria), presence of *café-au-lait* spots, thyroid palpation, bone deformities or asymmetry, target height (TH), bone age (BA) analyzed according to the Greulich & Pyle atlas (^16^), BA advancement (BA-CA), and predicted adult height (PAH) according to Bayley-Pinneau tables for average bone age (^17^).

The hormonal profile included basal and GnRH-stimulated luteinizing hormone (LH) and follicle-stimulating hormone (FSH) levels, which were measured via immunofluorometric assays (IFMA; Perkin Elmer; Cat# B031-101, RRID:AB_2783737 for LH and Cat# B017-21, RRID:AB_2783738 for FSH) before 2012 and via electrochemiluminescence assays (ECLIAs; Roche Diagnostics; CaT# 1732234, RRID: AB_2800498 for LH and Cat# 11775863, RRID:AB_2800499 for FSH) after 2012. The serum estradiol levels were measured via fluoroimmunoassays (FIA; Perkin Elmer; Cat# B056-101, RRID:AB_2927557) before 2012 and via ECLIA (Roche Diagnostics; CaT# 03000079, RRID:AB_2893079) after 2012. Basal LH levels were in the prepubertal range if they were < 0.6 IU/L (IFMA) or < 0.3 IU/L (ECLIA), whereas the GnRH and GnRH analog-stimulated LH peak cutoff levels were < 6.9 IU/L/< 5.0 IU/L (IFMA/ECLIA) and < 10 IU/L/< 5 IU/L (IFMA/ECLIA), respectively (^18^). Serum estradiol levels were in the pubertal range if they were > 25 pg/mL (FIA) or > 28 pg/mL (ECLIA).

Imaging studies included pelvic ultrasound performed in all patients. After the diagnosis of PPP of ovarian origin, all but two patients (patients 15 and 18) underwent bone scintigraphy to rule out FD.

During the treatment of PPP with distinct therapeutic agents (alone or combined), data on anthropometric parameters, growth velocity, BA, occurrence of vaginal bleeding, pelvic ultrasound, and occurrence of CPP were also documented.

The criteria for treatment discontinuation were a CA of 11-11.5 years, a BA of approximately 12-12.5 years, a PAH within the target height, and the perception of adequate psychosocial acceptance of puberty (^19^).

The duration of treatment, growth after cessation of treatment, adult height (AH), CA at menarche, menstrual cycle pattern, hormonal profile, and clinical follow-up data were also analyzed.

### Genetic diagnosis

DNA from the peripheral blood of 17 patients was analyzed via ddPCR. Two ddPCR mutation detection assays were used, each targeting the p.R201C or p.R201H mutation. For each assay, the TaqMan PCR system was used with two fluorescence probes: the first was labeled with HEX, which targets the wild-type (WT) allele, and the second was labeled with 6-FAM, which targets the mutated allele. The mixture (reagents + DNA) was divided into 20,000 nanodrops, which were placed on a PCR plate and amplified by PCR. After amplification, the fluorescence of each droplet was read on a 200 Droplet Digital PCR system (Bio-Rad) and was classified as positive (drop containing the target sequence) or negative. The results were analyzed using QuantaSoft software (Bio-Rad Laboratories) to determine the total number of positive droplets in the original sample. The results are reported in fractional abundance form, which corresponds to the percentage of mutated alleles out of the total number of alleles (ratio of mutated alleles/mutated + WT alleles) × 100. After validation, the established limit of detection was 0.1% fractional abundance.

### Statistical analyses

For descriptive statistics, categorical variables are expressed as percentages, and continuous variables are expressed as the means and SDs for variables with a normal distribution or as medians and interquartile ranges for variables with a nonnormal distribution. The Shapiro–Wilk test was used to determine normality. A paired t test was applied to compare continuous variables between related groups. The statistical analysis was performed on the R x 64 platform (v 4.1.0, 2021, Vienna, Austria). *P* values <0.05 were considered statistically significant.

## RESULTS

Among 18 female patients, 8 presented with the classic triad of MAS, and 10 presented with two clinical features of MAS. FD was confirmed in 11/16 (68.7 %) of the patients by bone scintigraphy, and *café-au-lait* skin spots were observed in 14/18 (77.8 %) of the subjects. Of those presenting with the classic triad, one patient had hyperthyroidism (patient 4, **Table 1**). The clinical data of all MAS patients are shown in **Table 1**.

**Table 1.** Clinical features of patients with peripheral precocious puberty of ovarian origin due to McCune-Albright syndrome at diagnosis

Patient	First sign of precocious puberty	CA at first sign (yr)	CA at first medical examination (yr)	Height (SDS)	Tanner stage (B/PH)	BA-CA	OFD	*Café-au-lait* skin spots	Other endocrine hyperfunctions
1	VB	2.7	3.1	0.3	2/1	0.4	+	+	-
2	Thelarche + VB	3	6.5	1.54	4/1	5.5	+	+	-
3	VB	2	10	-1.0	3/4	-1.1	+	+	-
4	Thelarche	0.6	5	NA	3/2	-	+	+	Hyperthyroidism
5	Thelarche + VB	3	5.2	4.15	3/1	4.8	+	+	-
6	Thelarche + VB	3	4.8	-0.57	4/2	2.8	+	+	-
7	Thelarche	1.5	3.7	2.15	4/1	1.2	+	+	-
8	Thelarche	NA	9.1	0.0	2/2	1.9	-	+	-
9	VB	3.7	4.6	0.7	2/1	4.3	+	-	-
10	Thelarche	5.7	6.7	0.1	4/2	2	-	+	-
11	Thelarche	4	6.6	3.0	3/2	4	+	-	-
12	Thelarche	3	4.1	2.27	4/1	2.1	-	+	-
13	Thelarche + VB	4.9	6.2	1.36	3/1	4.8	-	+	-
14	Thelarche	4.9	6.9	0.0	2/1	1	-	+	-
15	Thelarche + VB	4	5	1.82	2/1	0.7	NA	+	-
16	Thelarche	4	4	0.55	2/1	3.5	+	-	-
17	Thelarche +VB	6.1	6.1	0.75	2/1	0.6	+	-	-
18	VB	0.2	0.5	-0.16	3/1	1.3	NA	+	-

VB: vaginal bleeding; BA: bone age; CA: chronological age; B: breast development; PH: pubic hair; OFD: osteofibrous dysplasia; NA: not available

### Peripheral precocious puberty of ovarian origin

The mean CA at the onset of the first pubertal signs was 3.3 ± 1.6 years, whereas the first medical examination revealed a mean CA of 5.4 ± 2.1 years. Thelarche represented the first clinical sign in 8/18 patients (44.5%), followed by thelarche and vaginal bleeding in 6/18 (33.3%) and isolated vaginal bleeding in 4/18 (22.2%). However, at the first medical physical examination, all subjects presented with breast development (B2-B4 Tanner stage), while 6/18 (33.3%) also presented with pubarche (PH2-PH4) (**Table 1**). BA advancement greater than 1 year was identified in 13/17 (76%) subjects. The mean ΔBA-CA was 2.3 ± 1.9 years and ranged from -1.1 to 5.5 years. At diagnosis, PAH was significantly below the TH in 6/10 (60%) patients, with a mean height difference of -11.2 cm (95% CI -19.6, -2.7) (p = 0.015). **Table 1** summarizes the individual clinical features of these patients.

Basal LH was at prepubertal levels in 100% of the patients, and FSH was suppressed in 72.2% of the patients. GnRH-stimulation was performed in nine patients. The median GnRH-stimulated LH peak was 0.6 (IQ 0.2; 2.9) IU/L, while the FSH peak was 1.22 (IQ 1.0; 7.56) IU/L. The median serum estradiol concentration was 35.6 (IQ 23.6; 200.4) pg/mL and ranged from 10 to 783.2 pg/mL. Notably, four patients (22.2%) presented estradiol values in the prepubertal range.

At diagnosis, ovarian cysts were identified in 82.3% (14/17) of the patients at the first ultrasound exam: eleven patients had unilateral cysts, and 3 patients had bilateral cysts. The patients then underwent pelvic US, and sometimes cysts appeared in the other ovary. Individual imaging data and laboratory profiles are shown in **Table 2**.

**Table 2.** Laboratory and imaging profiles of patients with peripheral precocious puberty of ovarian origin due to McCune-Albright syndrome at diagnosis

Patient	LH (IU/L)		FSH (IU/L)		E_2_ (pg/mL)	Assay	Ovarian cyst	Laterality	Largest diameter cyst	Bone lesion sites on scintigraphy
	Basal	Peak*	Basal	Peak*						
1	<0.15	-	<0.6	-	35.3	ECLIA	+	Unilateral	LO; 37 mm	Craniofacial
2	<0.6	-	<1	-	783.2	IFMA	+	Bilateral	NA	Craniofacial
3	<0.1	-	<1	-	46	IFMA	+	Unilateral	RO; 26 mm	Craniofacial, right lower limb
4	<0.6	-	<1	-	290	IFMA	+	Unilateral	NA	Craniofacial, bilateral lower and upper limbs
5	<0.15	1.5	0.19	7.6	222.5	IFMA	+	Unilateral	RO; 27 mm	Craniofacial, left upper and lower limbs
6	<0.6	<0.6	<1	< 1	133	IFMA	NA	NA	NA	Left upper and lower limbs, left ribs
7	<0.1	0.2	<1	<1	13	IFMA	-	-	-	Right hemi body
8	<0.6	-	4.1	NA	31	IFMA	+	Unilateral	RO; 45 cm^3^	Normal
9	<0.6	3.4	<1	8.4	14	IFMA	+	Unilateral	RO, 45 mm	Right upper and lower limbs
10	<0.6	-	<1	5.8	34	IFMA	+	Bilateral	NA	Normal
11	<0.6	<0.6	<1	<1	36	IFMA	-	-	-	Right lower limbs
12	<0.6	-	<1	NA	9.3	IFMA	+	Bilateral	10 mm (multiples)	Normal
13	<0.1	-	0.3	NA	134.1	ECLIA	-	-	-	Normal
14	<0.1	2.9	1.3	-	31.2	IFMA	+	Unilateral	NA	Normal
15	0.02	0.2	0.01	1.1	598	IFMA	+	Unilateral	LO; 44 mm	NA
16	<0.1	0.1	<0.3	1.2	<10	IFMA	+	Unilateral	35 mm	Craniofacial, left lower limb
17	0.14	NA	<0.1	NA	35	IFMA	+	Unilateral	RO; 22 cm^3^	Craniofacial, lower limbs
18	<0.1	NA	<0.1	NA	463	IFMA	+	Unilateral	24 mm	NA

* After the GnRH/aGnRH stimulation test. IFMA: immunofluorometric assay; ECLIA: electrochemiluminescence assay; LO: left ovary; RO: right ovary; NA: not available

### Genetic diagnosis

Seventeen patients (8 with the classical triad and 9 with two clinical features of MAS) underwent genetic analysis of peripheral blood DNA samples via ddPCR. The ddPCR method was able to identify the presence of *GNAS* mutations in 9/17 (52.9%) patients: 4/8 (50%) patients with typical MAS and 5/9 (55%) patients with two MAS features. *GNAS* mutations, p.R201C or p.R201H, were identified in 2 and 7 samples, respectively. The fractional abundance ranged from 0.26% to 5.1% in the patients with typical MAS and from 0.1 to 5% in those with two MAS features (**Table 3**).

**Table 3.** Genetic diagnosis of MAS via ddPCR

Patient	ddPCR	
	Mutation detection	Fractional abundance
**Classical Triad (n = 8)**	**4/8 (50%)**	
1	R201C	1.2%
2	-	-
3	-	-
4	R201H	5.1%
5	R201H	1.2%
6	-	-
7	R201H	0.26%
15	-	-
**Two clinical features of MAS (n = 9)**	**5/9 (50%)**	
8	-	-
9	R201H	5.0%
10	NA	NA
11	R201H	0.66%
12	-	-
13	-	-
14	R201C	0.13%
16	R201H	1%
17	R201H	0.33%
18	-	-
Total (n = 17)	9/17 (52.9%)	

NA: DNA was not available for genetic study.

### Therapeutic management of PPP

In this cohort, PPP treatment was quite heterogeneous, which reflects the changes in MAS therapy over the years. The first choice of antiestrogen therapy was tamoxifen in 5 patients (27.8%) at a dosage of 10-20 mg/day, medroxyprogesterone acetate at 50-100 mg every 15 days in 3 patients (16.7%), aromatase inhibitors (letrozole 2.5 mg/day or anastrozole 1-2 mg/day) in 5 patients (27.8%), ketoconazole (200 mg/day) in 2 patients (11.1%), and unilateral oophorectomy in 2 patients (11.1%). Most patients (n = 10) were treated with drugs from at least two drug classes during the entire treatment period. Five patients were treated with monotherapy, two with tamoxifen (patients 10 and 12) and three with letrozole (patients 1, 16, and 18). One girl, who was 4 years of age (patient 15), did not receive any medical treatment due to spontaneous remission of pubertal signs.

Patients 7 and 13 were already receiving ketoconazole treatment at doses of 6 mg/kg/day and 8 mg/kg/day, respectively, at the time of referral to our service. Patient 7 switched to tamoxifen (10 mg/day), while patient 13 discontinued ketoconazole due to laboratory-detected adrenal insufficiency. Unfortunately, patient 13 was lost to follow-up before we adjusted the treatment plan.

Vaginal bleeding recurrence during clinical treatment was reported in 6/13 (46.1%) patients under anastrozole, letrozole or tamoxifen treatment either alone or in combination. Medroxyprogesterone acetate was introduced when prolonged vaginal bleeding occurred. According to available data, pubertal gonadotropic axis was diagnosed in 8/14 (57.1%) MAS patients through an increased basal or GnRH-stimulated LH peak in addition to progression of breast development. Two patients are currently younger than 8 years. Long-acting GnRH agonists (leuprorelin acetate 3.75 mg every 4 weeks or 11.25 mg every 12 weeks) were added to the treatment regimens of these patients (**Table 4**).

**Table 4.** Medical and surgical treatment, adverse effects and outcomes of patients with peripheral precocious puberty of ovarian origin due to McCune-Albright syndrome

Patient	Treatment	Secondary CPP	Adverse effects	VB during treatment	Duration of treatment (yr)	CA at the end of treatment (yr)	CA at menarche (yr)	Adult Height (cm/SDS)	Target Height (cm/SDS)
1	Letrozole	No	No	Yes	Under treatment	-	-	-	154.5/-1.2
2	TMX, Anastrozole, GnRHa	Yes	No	NA	4.2	11.3	12	152/-1.7	148.5/-2.2
3	MPA, TMX, GnRHa	Yes	No	NA	8	10	10.4	142/-3.3	157/-0.8
4	Left oophorectomy, Anastrozole, TMX, GnRHa	Yes	No	Yes	7	12	12.2	143.7/-3	NA
5	Anastrozole, MPA	NA	No	NA	NA	NA	NA	NA	163.5/0.21
6	TMX, Anastrozole, MPA	No	Hypertrichosis	Yes	7.1	NA	NA	152.6/-1.49	157.5/-0.78
7	Ketoconazole, TMX, GnRHa	Yes	Hypertrichosis	Yes	7.2	11	11.6	159/-0.5	NA
8	Right cystectomy Anastrozole or Letrozole	Yes	Laboratory hyperandrogenism	No	1.7	12	13.1	159/-0.5	156.7/-0.93
9	TMX, MPA	No	Hypertrichosis and uterine volume enlargement	Yes	6.2	11.4	13.6	149/-2.2	156.5/-0.95
10	TMX	NA	No	NA	Lost to follow-up	NA	NA	NA	169/1.13
11	MPA, Anastrozole, TMX, GnRHa	Yes	Edema	Yes	5.1	NA	12.1	160.8/-0.23	161/-0.2
12	TMX, GnRHa	Yes	No	No	5.1	11.3	12.4	167.5/0.8	159.5/-0.45
13	Ketoconazole	No	Adrenal insufficiency	No	Lost to follow-up	-	-	-	157/-0,8
14	MPA, TMX, GnRHa	Yes	No	No	4	11	NA	NA	NA
15	No treatment	NA	-	-	-	-	-	-	NA
16	Letrozole	NA	No	No	1.75	10	NA	-	NA
17	Right oophorectomy	No	No	No	-	-	13	165.5/0.4	169.5/0.9
18	Letrozole	No	No	No	Under treatment	-	-	-	NA

AI: aromatase inhibitors; TMX: tamoxifen; GnRHa: GnRH analog; MPA: medroxyprogesterone acetate; CPP: central precocious puberty; NA: not available; VB: vaginal bleeding; CA: chronological age

The main side effects reported were mild hypertrichosis in three patients under tamoxifen treatment (patients 6, 7 and 9), uterine volume enlargement in one patient during tamoxifen treatment (patient 9), mild biochemical hyperandrogenemia in one patient during treatment with an aromatase inhibitor (anastrozole 1 mg/day; patient 8), edema in one patient under medroxyprogesterone acetate treatment (patient 11), and laboratory-detected partial adrenal insufficiency in one patient under ketoconazole treatment (200 mg/day; patient 13). Tamoxifen treatment was discontinued in patient 9 due to uterine volume enlargement, and medroxyprogesterone acetate was continued as the sole therapy until the end of treatment. Cystectomy or oophorectomy was performed in three patients (patients 4, 8, and 16). Patient 4 underwent surgery prior to the initiation of clinical treatment at our hospital, while patient 8, who experienced pain and a high risk of torsion due to a large ovarian cyst (100 cm^3^), underwent surgery during anastrozole treatment. Following the surgery, patient 8 was switched to letrozole, which she tolerated well without any side effects. Patient 17 underwent early right oophorectomy because a complex cyst was detected via ultrasound. However, histopathological examination revealed no signs of malignancy.

The mean CA and BA at the interruption of treatment in 8 patients were 11 ± 0.7 years (range, 10-2.1 years) and 11.1 ± 1.5 year (range, 8.9-13 years), respectively. The available PAH value at the end of treatment (n = 6) was within the target height range in 4 patients (66%). The mean duration of treatment for precocious puberty was 5.0 ± 2.14 years (range, 1.7-8 years). Two girls (patients 1 and 18), with CAs of 8.4 and 2.5 years, respectively, are still receiving medical treatment (**Table 3**), while patient 13 was lost to follow-up after the diagnosis of partial adrenal insufficiency and ketoconazole withdrawal.

### Anthropometric and reproductive outcomes after treatment for PPP due to MAS

AH was reached by 10 patients and was within the TH range in six patients **(Table 3)**. The mean AH was 155.1 ± 8.7 cm (-1.17 SDS), whereas the mean TH was 158.2 cm (-0.6 SDS). After clinical treatment, the mean AH was 4.8 cm (95% CI, -6.5-16.1) higher than the mean PAH at baseline; however, the difference was not statistically significant (p = 0.34). In addition, the difference between the mean AH and TH was -2 cm (95% CI, -9.3-5.28), with no statistically significant difference (p = 0.52).

In 9 patients, menarche occurred at a mean CA of 12.2 ± 1.04 years (range, 10.4–13.6 years), while after treatment cessation, the mean CA was 0.9 ± 0.6 years **(Table 3)**. Regular menstrual cycles were detected in 7/9 patients (77.8%). The remaining two patients presented with an irregular menstrual cycle pattern, abnormal uterine bleeding, and intermittent suppression of serum LH and FSH levels, which likely indicates some degree of autonomous ovarian function. One patient (patient 11) became pregnant at 22 years of age; the entire pregnancy was uneventful, and she gave birth to a healthy child.

### Other features of MAS

Eleven of the 16 girls presented evidence of FD on bone scintigraphy with diphosphonate radiolabeling with technetium 99, 2 patients presented with isolated craniofacial involvement, and 9 patients presented with polyostotic involvement (**Table 2**). Patients 15 and 18 did not undergo imaging. Four girls (patients 3, 4, 7 and 11) had bone pathological fractures, all of whom were treated with antiresorptive agents. One patient (patient 7) developed optical pathway compression and airway obstruction and underwent decompressive surgery with satisfactory results. Pamidronate at a dose of 90 mg quarterly or zoledronic acid at a dose of 4 mg every six months was administered in seven patients for a mean duration of 3.7 years (range, 0.3-10.1 years) to treat persistent bone pain, with satisfactory results.

Patient 4 underwent orthopedic surgery on her right knee at age 10 in an attempt to halt right lower limb growth. This same patient was diagnosed with hyperthyroidism at 8.5 years of age. TSH levels were suppressed, and T3 and free T4 levels were increased. Thyroid scintigraphy revealed hyperuptake nodules in the right lobule. She was treated with methimazole for 6 months and subsequently underwent definitive treatment with radioiodine therapy.

## DISCUSSION

The diagnosis and therapeutic management of PPP in MAS patients can be challenging because it can affect short- and long-term outcomes. Our data confirmed that the natural history of PPP in girls with MAS is extremely variable, and its course is unpredictable, ranging from isolated vaginal bleeding and transitory thelarche to progressive PPP (^4^,^5^). In our current cohort, the first signs of PPP occurred at a very early age, which is concordant with previous studies (^6^,^20^). Breast development was the most common initial clinical sign, followed by vaginal bleeding. The laboratory profile of MAS patients indicated suppressed basal LH and FSH levels in most patients and that GnRH-stimulated LH peaked in the prepubertal range in all patients. Notably, some patients presented with prepubertal serum estradiol levels, which was likely due to blood sampling immediately after the resolution of vaginal bleeding. Similarly, estradiol levels were undetectable in 9/28 (32%) patients in a cohort of MAS patients (^15^). Therefore, in patients with clinical signs of estrogenic activity, sequential serum estradiol measurements should be recommended. Cumulative evidence shows the limitations of estradiol immunoassays. The determination of serum estradiol in pediatric patients should be performed at the initial evaluation and during treatment using methods with high sensitivity and specificity based on mass spectrometry (^21^).

Pelvic ultrasonography revealed ovarian cysts in most patients, the more common of which were unilateral cysts, as reported in previous studies (^22^,^23^). Interestingly, at diagnosis, some girls (patients 7, 10, and 13) did not exhibit ovarian cysts on ultrasound; however, the clinical and laboratory features of these girls allowed the diagnosis of gonadotropin-independent precocious puberty of ovarian origin. Other features, such as *café-au-lait* skin spots (patients 7, 13 and 18) and FD (patients 7 and 10), established the clinical diagnosis of MAS.

In this cohort, vaginal bleeding during treatment was reported by 6 patients, and medroxyprogesterone acetate effectively controlled recurrent vaginal bleeding in all these patients. However, we did not use this drug as a monotherapy because previous studies have shown no improvement in the growth rate or adult height (^11^,^12^,^22^). Ketoconazole treatment raised safety concerns, including adrenal insufficiency, as reported in previous studies (^24^,^25^).

The heterogeneity of pharmacological off-label treatments employed in girls with PPP and MAS is due to the lack of a gold standard. From 2003 to 2018, tamoxifen represented the first choice of treatment for PPP in patients with MAS in our endocrine unit; when tamoxifen was used as a monotherapy in 2 patients (10 and 12) and was combined with other agents in 7 patients, similar results to those reported in previous studies were obtained (^14^,^26^,^27^). Since 2018, letrozole has been the first-line treatment for PPP due to MAS, considering that most recent studies have demonstrated better long-term anthropometric outcomes regarding efficacy and safety (^15^,^28^,^29^).

The current MAS cohort experienced heterogeneous clinical treatments for PPP, and most girls were treated with a combination of drug classes. Therefore, attributing long-term responses to a single specific drug is challenging. Generally, the medical treatment of PPP in girls with MAS seems to be effective, with favorable outcomes observed in many cases. Surgical resection of ovarian cysts or oophorectomy is not usually recommended for the treatment of PPP due to concerns about the negative impact on fertility and the probability of cyst recurrence in the remaining ovarian tissue (^4^).

Although not mandatory for the overall MAS diagnosis, the genetic diagnosis of MAS plays a role in confirming the underlying genetic cause, mostly in cases where the classic triad is incomplete (^30^). In this study, we employed the ddPCR method to detect *GNAS* mutations in peripheral blood DNA samples, which resulted in a positive test in 52.9% of the entire patient group. Notably, 55% (5/9) of patients with 2 features of MAS presented a positive test. The genetic diagnosis of MAS does not modify the clinical and therapeutic approach of these patients but can contribute to active surveillance.

Despite the different treatment strategies, 60% of patients reached their adult height within the target range. Previous studies have compared predicted adult height (PAH) before and during treatment, as well as the bone age maturation rate, and have revealed improvements in these parameters following tamoxifen or letrozole treatment (^14^,^15^,^26^,^28^). One girl (patient 12) treated with tamoxifen monotherapy and GnRHa, having completed her growth, surpassed her target height. Importantly, no single antiestrogen treatment option has been definitively shown to be superior to other options.

The largest cohort of MAS patients evaluated for reproductive outcomes included 39 women with FD/MAS who experienced unfavorable gynecological events such as abnormal uterine bleeding (77%), hysterectomy (67%) and infertility (43%) (^31^). Conversely, our data revealed that, as of now, most patients experienced regular menstrual cycles, while only one patient (patient 11), who presented with PPP and FD, experienced spontaneous pregnancy. Notably, pregnancy did not alter FD symptoms in this patient.

Notably, PPP in boys with MAS is extremely rare, whereas macroorchidism is more commonly observed (approximately 44%) in males with MAS (^4^). Notably, testicular ultrasound and serial examinations are recommended for all males with MAS. Surgical intervention should be reserved for palpable, rapidly growing, or locally invasive lesions due to the potential risk of malignant degeneration in some cases (^4^). As with other forms of PPP in boys, medical management may include a combination of aromatase inhibitors (such as anastrozole or letrozole) and a competitive androgen receptor blocker (e.g., bicalutamide) or anti-androgen therapy (such as cyproterone acetate) (^4^).

FD is a skeletal disorder in which medullary bone is replaced by structurally unsound fibro-osseous tissue, since Gsα activation impairs the differentiation of skeletal stem cells (^3^). In our cohort, the commonly affected areas were craniofacial only in 2 patients, craniofacial and appendicular skeleton in 5 patients and appendicular skeleton only in 4 patients. Surgical intervention was required in 2 patients. FD lesions typically become established within the first few years of life, with 90% established by early adulthood (^3^). Intravenous pamidronate has beneficial effects, mainly in reducing pain in patients with FD (^3^).

Several studies have reported the beneficial effects of intravenous pamidronate and have revealed the ability of this nitrogen-containing bisphosphonate to improve bone turnover and decrease bone pain in adults and children with FD (^32^-^34^). A recent study revealed that 59% of patients with FD lesions in the appendicular skeleton experienced at least one lifetime fracture (^35^). Long-term follow-up of 11 MAS patients revealed that bisphosphonate use only reduced serum alkaline phosphatase and bone pain in 36% of patients after the onset of treatment (^36^). However, bone turnover markers were not systematically evaluated in the present study.

In conclusion, our study highlights the complex and variable nature of managing PPP in patients with MAS and demonstrates the need for individualized treatment approaches. Despite these challenges, the multifaceted treatment strategies employed have shown positive results in preserving adult height and reproductive function without significant side effects.

## Data Availability

All data generated or analyzed during this study are included in this manuscript. For further inquiries, please contact the corresponding author (V.N.B.).
